# "Getting your message through": an editorial guide for meeting publication standards

**DOI:** 10.1186/1757-7241-17-66

**Published:** 2009-12-23

**Authors:** Kjetil G Ringdal, Hans Morten Lossius, Kjetil Søreide

**Affiliations:** 1 Department of Research, Norwegian Air Ambulance Foundation, Drøbak, Norway; 2 Division of Emergency Medicine, Oslo University Hospital Ullevål, Oslo, Norway; 3 Faculty of Medicine, Faculty Division Ullevål University Hospital, University of Oslo, Oslo, Norway; 4 Department of Surgical Sciences, University of Bergen, Bergen, Norway; 5 Department of Surgery, Stavanger University Hospital, Stavanger, Norway

## Getting your message through

"Getting your message through" is the *sine qua non *of medical science. If you cannot make your peers understand your message, then you are bound to have manuscripts rejected or to have difficulties getting your valuable research published, read, and perused. The challenge for the scientist clinician is to present readable papers of general interest for judgment by editors, editorial board members, and peer reviewers while soundly describing the methods in a way that is recognizable to any knowledgeable scientist. Furthermore, the editors and governors of any Journal have the responsibility to ensure that the contributors' work receives the highest possible visibility by ensuring proper indexing and tracking.

The Scandinavian Journal of Trauma, Resuscitation and Emergency Medicine (SJTREM) is a peer-reviewed international journal indexed in PubMed, PubMed Central, Scopus, and Google Scholar. In addition, SJTREM has recently been accepted for indexing by MEDLINE. Decisions on tracking in EMBASE and by Thomson Reuters will occur shortly. The Journal is directed towards all health professionals involved in pre- and in-hospital emergency medicine and surgery, critical care, and trauma management. While SJTREM was primarily intended for a Scandinavian and Northern-European audience [1], it has received international attention and the journal deliberately works to increase its international attention [2]. The Journal follows the standards put forward in the 'Uniform Requirements for Manuscripts Submitted to Biomedical Journals' from the International Committee of Medical Journal Editors (ICMJE) [3], and is a member of the Committee on Publication Ethics (COPE) [4]. The editors have established and will continue to establish agreements with international clinical experts and researchers to provide subject-specific reviews.

## The strength of evidence

High quality scientific papers begin with a sound research design [5-7]. In addition to presenting the data and providing analysis of the results, the data must be subjected to statistical analysis using precise and correct methods [8-10].

Studies in trauma, resuscitation, and emergency medicine typically deal with interventions, but studies on prevention and aetiology, diagnosis and prognosis, and economic considerations are also relevant. For interventional trials, four attributes define the strength of a study's evidence. First, the ***level of evidence ***is attributed to the type of study design used (Tables 1 and 2) [11]. Second, the ***quality of the evidence ***is assessed by the presence or lack of bias (Table 3) [5]. The third attribute is ***statistical precision***, which is the ability of an analysis to distinguish true effects from effects merely resulting from random chance. Fourth, the ***choice of the study endpoint ***used to measure the effect depends on both the clinical value of that endpoint and the magnitude of the observed effect. As a given study may have strengths and weaknesses among these parameters, we will address some basic requirements that we look for in any study submitted to the Journal.

**Table 1:** The Oxford Centre for Evidence-based Medicine Levels of Evidence.

Level	Therapy	Diagnosis
**1a**	Systematic review (SR) of Randomised Clinical Trials (RCT)	SR of Level 1 diagnostic studies; Clinical Decision Rule (CDR) with 1b studies from different clinical centres.
**1b**	Individual RCT (with narrow confidence interval)	Validating cohort study with good reference standards, or CDR tested within one clinical centre.
**1c**	All or none^††^	Diagnostic finding whose specificity is so high that a positive result rules-in the diagnosis. Diagnostic finding whose Sensitivity is so high that a Negative result rules-out the diagnosis.
**2a**	SR of cohort studies	SR of Level 2a-c diagnostic studies
**2b**	Individual cohort study (including low-quality RCT; e.g., follow-up of <80% of patients)	Exploratory cohort study with good reference standards; CDR after derivation, or validated only on split-sample or databases
**2c**	Audits or "outcomes" research; Ecological studies	
**3a**	SR of case-control studies	SR of Level 3b and better studies
**3b**	Individual Case-Control Study	Non-consecutive study; or one without consistently applied reference standards
**4**	Case-series (and poor-quality cohort and case-control studies)	Case-control study, poor or nonindependent reference standard
**5**	Expert opinion without explicit critical appraisal, or based on physiology, bench research or 'first principles'	

Note: †† Met when all patients died before the Rx became available, but some now survive on it; or when some patients died before the Rx became available, but none now die on it.

The table is adapted and published with permission from the Oxford Centre for Evidence-based Medicine [11].

**Table 2:** Grades of Recommendation.

Grades	Study design
**A**	Consistent level 1 studies
**B**	Consistent level 2 or 3 studies ***or ***extrapolations from level 1 studies
**C**	Level 4 studies ***or ***extrapolations from level 2 or 3 studies
**D**	Level 5 evidence ***or ***troublingly inconsistent or inconclusive studies of any level

The table is published with permission from the Oxford Centre for Evidence-based Medicine [11].

**Table 3:** Major types of study bias in clinical research.

Bias	Source of error
Selection bias	Sample distorted by selection process
Information bias	Misclassification of the variables
Confounding bias	An extraneous variable that accounts for the observed result rather than the risk factor of interest

## Requirements for describing the study design

The most important phase of any research project is the planning and study design phase. Errors and shortcomings in this phase can have major negative impacts on the validity and reliability of the results. Incomplete and unsatisfactory reporting of research impedes the assessment of the strengths and weaknesses of studies reported in international scientific journals. The SJTREM requires a reader-friendly presentation of specific objectives, including any prespecified hypotheses, as well as information on the study's population, eligibility criteria for participants, and the setting and locations where the data was collected. Additionally, the frequency and cause of missing data should be reported along with a description of how missing data was handled.

The authors are required to present the statistical methods and tests used. In order to increase the reporting standards, SJTREM supports the use of the widely adopted Vancouver guidelines, which require authors to "*describe statistical methods with enough detail to enable a knowledgeable reader with access to the original data to verify the reported results*" [8]. For instructions on how to report statistical results in research manuscripts, we recommend using some of the more comprehensive books on the topic [7,12,13].

## Suggested guidelines for reporting biomedical research

The Editors of the SJTREM recommend that authors follow specific guidelines for reporting biomedical research studies available at the EQUATOR Network website [14]. In particular, commonly available guidelines and checklists should be followed when reporting randomized controlled trials (CONSORT statement) [15], systematic reviews (PRISMA statement) [16,17], diagnostic accuracy studies (STARD statement) [18], and observational research (STROBE statement) [19]. Articles concerned with improvement in healthcare quality can follow the SQUIRE guidelines [20,21]. Meta-analyses of randomized trials must adhere to the guidelines outlined in the PRISMA statement, while meta-analyses of observational research must adhere to the MOOSE statement guidelines [22,23]. The intentions of these statements are to identify the aspects of various research designs that must be reported to permit an adequate evaluation of the research [12].

## Where should the information be presented?

Readers and editors of a paper need to know what was **planned **(and what was not), what was **done**, what was **found**, and what the results **mean**. Hence, we suggest structuring the text of original research articles into headings summarised by the mnemonic acronym IMRAD (**I**ntroduction, **M**ethods, **R**esults, **A**nd **D**iscussion) [8,13,24]. This basic structure is common to standard scientific papers, although there may be minor variations.

The introduction or background section should be brief, but to the point, and should tell readers why the study was undertaken [13]. A straightforward way to structure the introduction would be to separate it into three parts: first, describe what is known and/or what the accepted status of the current topic is; second, describe what is unknown and what should be examined more closely; and finally, describe the aim(s) and purpose(s) of the study [25].

The methods section answers the questions **"who, what, why, when, and where?" **This section should include information regarding how the study was planned and designed, the included patient population, and how the sample was selected/recruited. Additionally, the means used to gather information must be presented along with the statistical methods and the statistical package used for analysis.

In the results section, authors should report the actual study sample (number of study subjects that were eventually included or participated). If necessary, this information can be presented in a flow chart. Additionally, authors should describe the outcomes and results, addressing one topic per paragraph, and working from the most important topic to the least important topic. Remember that the results should be presented objectively, without any qualitative words, evaluations, or interpretations; these statements belong in the discussion section. Tables and illustrations are meant as a supplement to the text, and the fewest possible number of tables and illustrations should be used. Data in tables and figures should be presented in the manner that results in the most clarity. One point worth stressing is that authors should avoid including tables with large amounts of data, as readers may find these too busy and difficult to read. All tables and figures should be comprehensible without referring to the text, and should have titles that are succinct and sufficiently self-explanatory [24].

The discussion section should begin with a clear and unambiguous summary of the key findings in no more than two or three sentences. Next, it must include a discussion of the strengths and weaknesses of the study. This should be followed by a comparison of the results with those from other published work. Only the major relevant works that confirm and contradict the findings should be cited. Next, this section should discuss what the study might indicate, explain the findings, and describe whether and how the study has increased the understanding of the field. Finally, authors should discuss what questions remain unanswered and suggest future work in the area [13,25,26].

The SJTREM strives to publish articles that are timely, credible, and enjoyable to read. We aim to publish original, important, and well-documented peer-reviewed research articles. The Editors expect authors to follow good standards and ethics for publication, and will take action regarding any report or detection of fraudulent research or scientific misconduct. We encourage authors to use SJTREM as a publication venue for reviews and original research papers in pre- and in-hospital emergency medicine and surgery, critical care, and trauma management, and we encourage authors to follow these guidelines for study design and presentation to ensure high quality scientific communication.
